# Level of Abdominal Aorta Bifurcation and Its Relation to the Ureter: A Radiological Study with Clinical Implications

**DOI:** 10.3390/diagnostics15172167

**Published:** 2025-08-26

**Authors:** Mohammed Al-Hajri, Ali Abduwani, Ilyas Al-Saadi, Nasser Al Sidairi, Mahmood Salim Nasser Al Riyami, Saleh Baawain, Srijit Das

**Affiliations:** 1 Department of Human & Clinical Anatomy, College of Medicine & Health Sciences, Sultan Qaboos University, Muscat 123, Oman; s136412@student.squ.edu.om (M.A.-H.); s136668@student.squ.edu.om (A.A.); s138387@student.squ.edu.om (I.A.-S.); 2 Radiology Program, Oman Medical Specialty Board, Muscat 132, Oman; nsm96266@gmail.com; 3 Department of Radiology & Molecular Imaging, Sultan Qaboos University Hospital, Muscat 123, Oman; mahmoodriy@hotmail.com (M.S.N.A.R.); ssab@squ.edu.om (S.B.)

**Keywords:** abdominal aorta, anatomy, CT scan, common iliac arteries, radiology, relation, vertebral level

## Abstract

**Background/Objectives:** The abdominal aorta (AA) bifurcates at the level of the L4 vertebra, giving origin to the right and left common iliac arteries (CIA). The CIA then bifurcates into external iliac arteries (EIA) and internal iliac arteries (IIA). The present radiological study aimed to (i) measure the diameter of the right and left CIA, (ii) measure the distance between the AA bifurcation and the point where the ureter crossed the ipsilateral iliac vessels, (iii) examine the angle of AA bifurcation, and (iv) observe the vertebral level at which the AA bifurcated. **Methods:** The retrospective cross-sectional study included contrast-enhanced CT angiograms of 200 patients (*n* = 144 males and 56 females) who attended the radiology department from 1 January 2022 to 31 December 2023. Two independent radiologists interpreted the angiograms. The results were interpreted using parametric and non-parametric statistical tests. **Results:** The mean angle of the AA bifurcation was 40.46° and 44.68° in males and females, respectively (*p* = 0.013). The mean diameter of the right and left CIA was wider in males than in females, but no statistical significance was found. The average distance between the AA bifurcation and the point where the right and left ureter crossed the ipsilateral iliac arteries was longer in females (60.99 and 60.29 mm, respectively) than in males (59.05 and 59.95 mm, respectively), but no statistical difference was found (*p* > 0.05). The most common vertebral level for AA bifurcation was the L4 vertebra, which was found in 137 (68.50%) patients. The AA bifurcated at the level of L3 and L5 in 52 (26.00%) and 11 (5.50%) patients, respectively. **Conclusions:** Understanding the radiological anatomy of the CIA and AA bifurcation and its relation to the ureter is crucial for surgeons performing aortic and pelvic surgeries.

## 1. Introduction

The abdominal aorta (AA) provides the major blood supply to the abdomen, pelvis, and lower limbs. It is the continuation of the descending aorta as it enters the abdominal cavity from the aortic hiatus of the diaphragm at the level of the T12 vertebra [1]. It gives rise to multiple unpaired and paired branches. The terminal branches of the AA are the right and left common iliac arteries (CIA). The right and left CIA arise at the L4 vertebral level and travel downward and laterally along the medial edge of the psoas muscle. Both the CIA end in front of the sacroiliac joint by dividing into external and internal iliac arteries (IIA) [2]. The IIA is one of the arteries that enter the pelvis. It goes to the upper margin of the greater sciatic foramen and divides into anterior and posterior divisions; both divisions give branches that supply the perineum, the pelvic wall, the pelvic viscera, and the gluteal region [3]. The external iliac artery (EIA), on the other hand, follows the pelvic brim and gives rise to the deep circumflex iliac branches and the inferior epigastric artery. It leaves the false pelvis by traveling under the inguinal ligament to become the common femoral artery, which supplies the lower limb [4].

The AA bifurcation is a common place of atherosclerosis, which can lead to symptoms such as critical limb ischemia [5]. Thus, it is essential to understand the normal level at which the CIA originates, the angle of AA bifurcation, and the diameter of the CIA at the bifurcation point of the AA. It was found that there is a positive correlation between aortoiliac atherosclerosis and the angle of AA bifurcation [6]. Moreover, a relationship exists between high blood pressure in males and the diameter of the CIA, whereas in females, only diastolic blood pressure is associated with the diameter [7]. Understanding the normal variations in the diameter of the CIA can help differentiate between normal and pathological conditions. Furthermore, ethnicity should be considered, as some have smaller CIA diameters, such as Chinese, when compared to other ethnicities [8].

The ureters are bilateral tubes with diameters of 3–4 mm that extend from the kidneys into the urinary bladder. The ureters descend inferiorly in the retroperitoneal space, pass anterior to the psoas major muscle, enter the pelvis at the point of CIA bifurcation, and continue along the posterolateral pelvic wall, and finally enter the bladder via the trigone [9]. During its course, the ureters show three strictures. The first stricture is formed at the ureteropelvic junction, where the renal pelvis continues as the ureter. The second stricture is formed as the ureter crosses the pelvic brim, at the level of the sacroiliac joint, which is the level where the CIA usually divides into the IIA and EIA. The third stricture is formed as the ureter enters the bladder at an acute angle [10]. These sites are of great importance, as calculi might dislodge in any of the three constrictions. Due to the anatomical structure, the ureter closely resembles the blood vessels. Hence, anatomical knowledge is needed for performing pelvic surgeries like salpingectomy, as the ureter is closely related to the blood vessels in that region. Failure to differentiate between the vessels and the ureter may result in accidental clamping.

It is important to study the anatomical relation of the ureter to the lower abdominal and pelvic vessels. The previous literature explored the relation between the ureter and the CIA bifurcation [11]. An extensive search of the literature did not reveal any published literature on the relation between the bifurcation of the AA and the point at which the ureter crosses the iliac vessels. The knowledge of the normal diameter of the CIA is important for the diagnosis of vascular pathologies such as aortic aneurysms and stenosis. Furthermore, the ureter may be at risk of injury during aortic aneurysm repair surgeries. Hence, it is important to study the anatomical relationship between the AA and the ureters.

The present study aimed to examine the variation in the diameter of CIA proximal to its bifurcation and compare it among genders and different age groups (≤26 years, 27–34 years, 35–42 years, and ≥43 years), measure and examine the angel and vertebral level at which the AA bifurcated and compare it among genders, and measure the distance between the AA bifurcation and the point at which the ureter crosses the iliac arteries and compare it among genders and age groups.

## 2. Materials and Methods

The retrospective cross-sectional study utilized the contrast-enhanced CT angiograms of 200 patients (*n* = 144 males and 56 females), who attended the radiology department at Sultan Qaboos University Hospital in the period from 1 January 2022 to 31 December 2023.

### 2.1. Inclusion

All patients who attended the radiology department in Sultan Qaboos University Hospital from 1 January 2022 to 31 December 2023, aged between 19–50 years, with no history of any vascular pathology such as aneurysms or atherosclerosis, and those who did not experience any form of abdominal trauma which could have impacted the vascular dimensions were included in the study.

### 2.2. Exclusion

All patients who were younger than 19 years, older than 50 years, or those who suffered from any form of vascular pathologies that were known to directly change the vascular anatomy and dimensions, or abdominal trauma, were excluded from the study. After applying the inclusion and exclusion criteria, a total of 200 patients were shortlisted.

### 2.3. Radiological Investigation

The contrast-enhanced angiograms were interpreted by two radiologists from the Sultan Qaboos University Hospital, and the data were collected in a spreadsheet. The maximal diameter of the right and left CIA was calculated from the outer-to-outer layer as reported in an earlier study [12]. To determine the vertebral level, the iliac crest was located first, and it was used as a reference point to identify the rest of the vertebral column, and the level of the AA bifurcation was recorded. The angle of the AA bifurcation and the distance from the AA bifurcation to the point where the ureter crossed the iliac arteries were recorded in a coronal view.

### 2.4. Data Analysis

The analysis was done by SPSS software (version 26). To determine whether the diameter, angle, and distance follow a normal distribution, a one-sample Kolmogorov–Smirnov (K-S) test was performed. If the *p*-value was more than 0.05 (*p* > 0.05), the variable was normally distributed. On the other hand, a *p*-value of 0.05 or less (*p* ≤ 0.05) indicates that the data did not follow the normal distribution. If the variable followed the normal distribution, the independent *t*-test and one-way ANOVA test were used to compare the mean value among genders and age groups, respectively. On the other hand, if the variable was found not to follow the normal distribution, the Mann–Whitney U test and the Kruskal–Wallis test were used to compare the mean value among genders and age groups, respectively.

## 3. Results

Following a one-sample K-S test, the diameter of the left CIA did not show a normal distribution. The diameter of the right CIA, the angle of AA bifurcation, and the distance between the AA bifurcation and the point where the right and left ureters crossed the ipsilateral CIA were found to follow a normal distribution.

**Figure 1:**
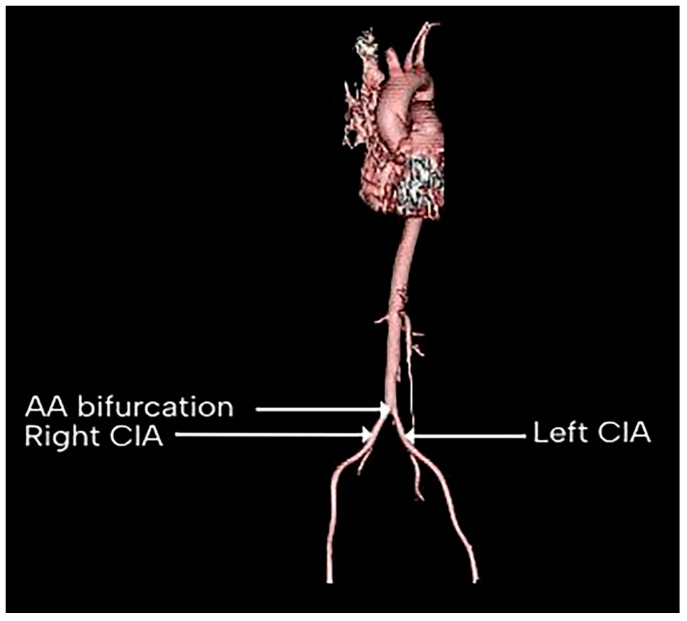
Three-dimensional image showing the AA bifurcation into the right and left CIA. AA: abdominal aorta, CIA: common iliac artery.

**Figure 2:**
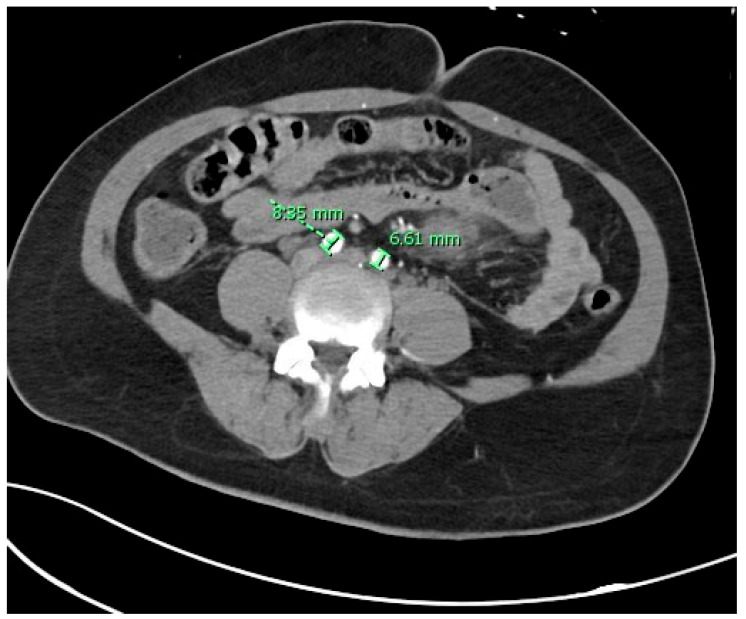
CT scan showing the measured diameter of the right and left CIA. CIA: common iliac artery.

Figure 1 shows a 3D image of the AA bifurcation, giving rise to the right and left CIA. The mean diameter of the right CIA proximal to its bifurcation point in 200 patients was found to be 9.08 ± 1.22 mm. The mean diameter in males (*n* = 144 males) was found to be 9.17 ± 1.13 mm, while in females (*n* = 56 females), the mean diameter of the right CIA at the point proximal to its bifurcation was 8.85 ± 1.42 mm. The difference in the diameter of the right CIA proximal to the artery’s bifurcation between males and females was found to be statistically insignificant (*p* > 0.05). Figure 2 shows a CT scan with the measured diameter of the right and left CIA.

The mean diameter of the left CIA, proximal to its bifurcation point, in 200 patients was found to be 9.12 ± 1.18 mm. The mean diameter of the left CIA proximal to its bifurcation in males and females was found to be 9.18 ± 1.09 mm and 8.96 ± 1.36 mm, respectively. The difference in the diameter of the left CIA proximal to the artery’s bifurcation between males and females was found to be not significant (*p* > 0.05). Table 1 shows the mean diameter of the right and left CIA proximal to the point of bifurcation among the four age groups.

**Figure 3:**
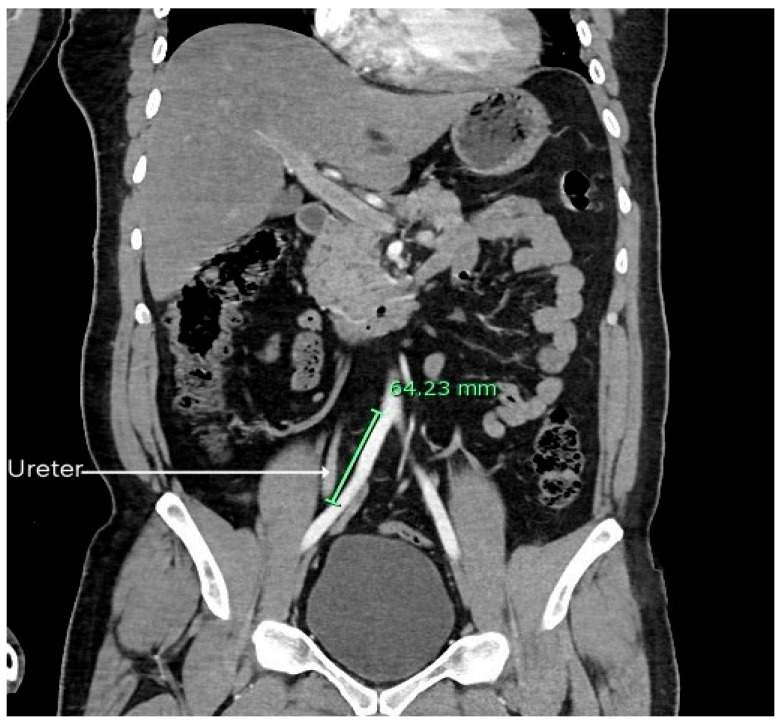
CT scan showing the measured distance between the AA bifurcation and the point where the left ureter crossed the left iliac vessels.

The mean distance between the bifurcation of the AA and the point where the right ureter crossed the right iliac arteries in 200 patients was found to be 59.59 ± 12.79 mm. The mean distance between the AA bifurcation and the point where the right ureter crossed the right iliac arteries in males and females was found to be 59.05 ± 11.45 mm and 60.99 ± 15.75 mm, respectively. However, the difference between the two genders was insignificant (*p* > 0.05). Figure 3 shows the measured distance between the AA bifurcation and the point where the left ureter crossed the left iliac arteries.

The mean distance between the bifurcation of the AA and the point where the left ureter crossed the left iliac arteries in 200 patients was found to be 60.05 ± 11.38 mm. The mean distance between the AA bifurcation and the point where the left ureter crossed the left iliac arteries in males and females was found to be 59.95 ± 10.59 mm and 60.29 ± 13.32 mm, respectively. The difference between males and females was found to be statistically insignificant (*p* > 0.05). Table 2 shows the mean distance between the AA bifurcation to the point where the ureter crossed the ipsilateral iliac arteries.

**Figure 4:**
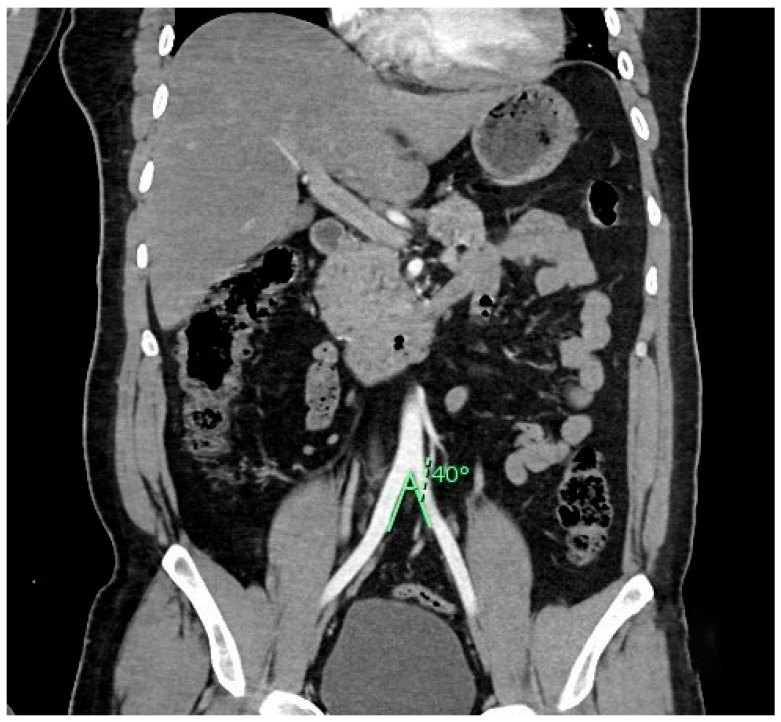
Coronal CT section showing the measurement of the AA bifurcation. AA: abdominal aorta.

The mean angle of AA bifurcation in 200 patients was found to be 41.77° ± 10.34°. The mean angle of AA bifurcation in males and females was found to be 40.64° ± 9.77° and 44.68° ± 11.25°, respectively. The difference between males and females was found to be statistically significant (*p* = 0.013). However, the effect size was found to be small (Cohen’s d = 0.38). Therefore, even though the difference was significant (*p* = 0.013), the magnitude of the difference was minimal. Figure 4 shows a coronal section of the abdomen with the measured angle of the AA bifurcation.

**Figure 5:**
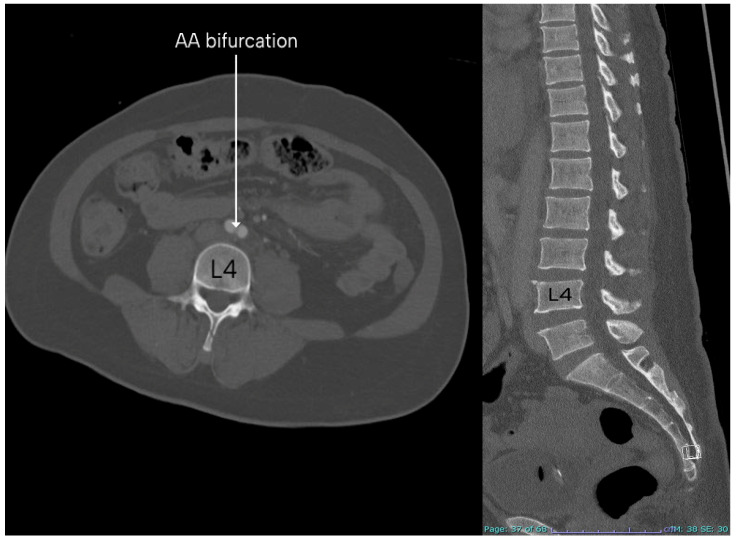
Two CT sections of the same patient. The axial section shows the bifurcation of the AA, and the vertebral level can be observed in the sagittal section. AA: abdominal aorta.

**Figure 6:**
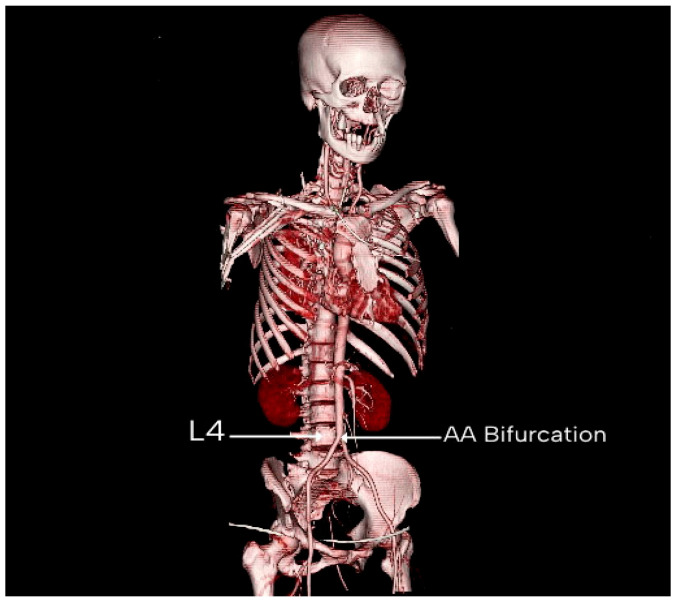
Three-dimensional reconstructed image in which the AA bifurcation is seen at the level of the L4 vertebra. AA: abdominal aorta.

The most common vertebral level at which the AA bifurcated was L4, which was the case in 137 patients (68.50%). Following that, the AA bifurcated at the level of L3 in 52 patients (26.00%). In the other 11 patients (5.50%), the AA bifurcated at the level of the L5 vertebra. Table 3 shows the level of AA bifurcations in males and females. Figure 5 shows two CT sections showing the bifurcation of the AA and the vertebral level of bifurcation. Figure 6 is a 3D reconstructed image showing the AA bifurcation at the level of the L4 vertebra.

## 4. Discussion

The development of the cardiovascular system begins in the middle of the third week of intrauterine life. The arterial system begins as the aortic arches and the paired dorsal aorta, which unite to form the descending aorta, which extends the whole length of the embryo. It gives several branches that supply the thoracic region, such as the intercostal arteries, and it supplies the abdominal region by giving lateral and ventral branches. Lateral branches supply the kidney, gonads, suprarenal glands, and the posterior wall by the lumbar branches [13]. The ventral branches are divided into the celiac trunk, the superior mesenteric artery, and the inferior mesenteric artery. The first ventral branch is the celiac trunk, which supplies the foregut derivatives such as the esophagus, stomach, liver, gallbladder, biliary system, pancreas, and the first part of the duodenum proximal to the opening of the bile duct [14]. The second ventral branch gives rise to the superior mesenteric artery, which supplies the midgut derivatives, which extend from the ampullary region of the second part of the duodenum into the proximal two-thirds of the transverse colon [15]. The last ventral branch of the AA is the inferior mesenteric artery. It supplies the hindgut derivatives, which are the distal third of the transverse colon, the descending colon, the sigmoid colon, and the proximal two-thirds of the rectum [16]. Moreover, the AA bifurcates and gives rise to the terminal branches, which are the CIA. The CIA developed from the fifth lumbar artery. Finally, the median sacral artery persists as a remnant of the dorsal aorta [17].

There are many types of arteries. All the arteries have three main layers: the tunica intima, tunica media, and tunica adventitia. The first type is the elastic arteries, which consist of the tunica intima, which has a thin endothelium inner layer of the vessel. Also, it contains tunica media, which is composed of smooth muscle and a high number of elastic fibers. The tunica media is considered the thickest part, allowing the expansion and relaxation of the artery in every phase of the cardiac cycle. The outermost layer is the tunica adventitia, which is composed of collagen, elastic fibers, and minute blood vessels that are called vasa vasorum. Moreover, there is an elastic layer that separates the tunica media from the tunica adventitia, which is termed the external elastic lamina. The aorta, pulmonary trunks, and CIA are examples of elastic arteries [18,19,20]. The second type is the muscular arteries, which consist of the same three layers but with a different composition. The tunica intima consists of polygonal endothelium with subendothelial connective tissue and a small amount of longitudinal smooth muscle. The tunica media contains many layers of circularly arranged smooth muscle cells, which help in maintaining and regulating blood pressure. The tunica adventitia consists of very thick collagen and elastic fibers with few longitudinal smooth muscles, and it also contains vasa vasorum. Moreover, there are two elastic layers: the internal elastic lamina, which is present between the tunica intima and the tunica media, and the external elastic lamina, which is present between the tunica media and adventitia. Major examples of muscular arteries are the carotid and the femoral arteries [20]. The last type of artery is the arteriole. In arterioles, the tunica intima consists of endothelium and subendothelial connective tissue. The tunica media contains three layers of spirally arranged smooth muscle cells, and the tunica adventitia is composed of collagen and elastic connective tissue. The internal elastic lamina and external elastic lamina are usually present in large arterioles but are absent in smaller arterioles. CIA has a thinner wall compared to the aorta, and they differ in the tunica media, which suggests that CIA might not possess the typical characteristics of elastic arteries [20,21].

The mean diameter of the CIA may vary between males and females. A study conducted in China by Hu et al. (2022) included contrast-enhanced CT angiograms of 625 patients, with a median age of 60 years (*n* = 380 males and 245 females), and reported that the mean diameter of the right CIA was found to be 10.70 mm [22]. Among males, the diameter was found to be 11.20 mm, while among females, it was found to be 9.75 mm. The mean diameter of the left distal CIA was 10.60 mm in the same study. Among males, the diameter of the left CIA was 11.20 mm, while in females, it was 9.72 mm. The study reported the difference in diameter between males and females to be significant (*p* < 0.001) [22].

A meta-analysis based on 5785 cases conducted by Koziej et al. (2025) [23] included 258 studies from 1978 to 2022, and finally, 26 studies were selected for the analysis. The mean diameter of the right CIA was found to be 10.53 mm, while the mean diameter of the left CIA was found to be 10.16 mm. Regarding genders, in males, the diameter of the right and left CIA was found to be 11.24 and 10.79 mm, respectively. On the other hand, the right CIA in females was 9.61 mm, while the left CIA was 9.34 mm. However, the study reported that the difference in diameter of the right and left CIA between males and females was not significant [23]. In the present study, the diameter of the right and left CIA was wider in males compared to females, i.e., 9.17 and 9.18 mm vs. 8.85 and 8.96 mm, respectively. The results of this study are comparable to the previous reports [22,23], where the diameters of the right and left CIA were also found to be wider in males than in females. However, in the present study, the difference was not statistically significant (*p* > 0.05).

In an earlier meta-analysis, it was reported that there was a relation between the diameter of the CIA and age [23]. The age group of 60–70 was found to have enlargement of the left CIA diameter, while the age group older than 70 years had an enlargement of the right CIA diameter [23]. A study conducted by Góes et al. (2020) in Brazil focused on the relation between age and diameter of CIA, including the abdominal CT angiograms of 157 patients (*n* = 69 men and 88 females) [24]. The rate of diameter enlargement with age was reported to be significant in males (*p* = 0.02). On the other hand, in females, there was no significant difference between the diameter and age (*p* > 0.05) [24]. The diameter of the CIA is important for the hemodynamics of the blood vessel. Knowledge of the normal vascular diameter is important for the diagnosis of an aneurysm. Early detection of aneurysms by screening is associated with a lower mortality rate [25]. Interestingly, the length of the blood vessel may also be important, and a previous study stated that the Asian population tends to have shorter CIA when compared to Caucasians [26].

To the best of our knowledge and thorough search of the literature, we did not find any studies on the distance from the AA bifurcation to the point where the ureter crossed the iliac arteries. However, a study conducted in China by Han et al. (2021) [11] involving CT angiograms and urograms of 116 female patients focused on the distance between the CIA bifurcation and the point where the ureter crossed the iliac arteries. The distance from the right CIA bifurcation to the point where the right ureter crossed the right iliac arteries was found to be 12.4 mm. On the left side, the distance was found to be 8.8 mm [11].

Knowing the variation in the distance between the AA bifurcation to the point where the ureters cross the iliac arteries (CIA or EIA) is important during the preoperative plan, especially before dissection or graft placement procedures, as it can cause iatrogenic injury to the ureter [27]. Moreover, this distance can be easily seen in cases of renal calculus and ureteric obstruction, as the urine will accumulate in the ureter and cause dilation of the ureter that can be unilateral or bilateral [28].

The difference in the angle of AA bifurcation has an unknown impact on the body till now [29]. Many studies focus on the variations of the AA bifurcation angle. A study conducted by Shakeri et al. (2007) involved angiograms of 59 patients aged over 40 years (*n* = 48 males and 11 females) [6]. Among the 59 patients, 33 were identified as normal individuals, while others had an aortoiliac occlusive disease. Among normal individuals, the mean angle of AA bifurcation was 34.6°. The study reported no significant difference between the angle of AA bifurcation and gender [6].

A study conducted in Thailand by Lakchayapakorn et al. (2008) discussed the angle of AA bifurcation among 65 cadavers (*n* = 37 males and 28 females) aged between 50 and 90 years [30]. The mean AA bifurcation angle was reported to be 54° [30]. The angle of aortic bifurcation in males and females was reported to be 55° and 53°, respectively. The study reported no significant difference between the angle of AA bifurcation and the genders (*p* > 0.05) [30]. Another cadaveric study conducted in India by Deswal et al. (2014) included 25 cadavers (*n* = 16 males and 9 females) and reported that the mean angle of abdominal bifurcation was 50.16° [31]. The mean angle was found to be 49.37° and 51.55° in males and females, respectively. The study also reported no significant difference between the angle of AA bifurcation and gender (*p* > 0.05) [31]. In the present study, it was found that the angle of AA bifurcation was wider in females compared to males, 44.68° and 40.64°, respectively. Even though the difference in the angle of bifurcation between males and females was found to be significant (*p* = 0.013), the magnitude of the difference was found to be minimal (Cohen’s d = 0.38).

Many studies have found that as humans age, the bifurcation of AA moves downward. The reduction of the spinal cord is due to the loss of elasticity of the intervertebral disc, which will cause changes in the dynamics of the individual. Moreover, osteoporosis and osteopenia also contribute to the reduction of the spinal cord, resulting in caudal displacement of the AA bifurcation [6,30]. A study conducted in Jordan by Khader et al. (2022) included the contrast-enhanced CT angiograms of 100 patients (*n* = 50 males, *n* = 50 females) aged 20–50 years [32]. The most common vertebral level of AA bifurcation was the L4 vertebra in 65 patients (65.00%), while the least common vertebral level was L4/L5 in 1 patient (1.00%) [32]. Another study conducted in Thailand by Lakchayapakorn et al. (2008) discussed the AA bifurcation position in relation to the lumbar vertebrae among 65 cadavers (*n* = 37 males and 28 females) aged between 50 and 90 years [30]. The AA bifurcation was found to be between L3 and L5, with the most common vertebral level of the AA bifurcation being L4 in 41 cases (63.00%), while the least common vertebral level of AA bifurcation was L3 in 2 cases (3.00%) [30]. Moreover, in the United States of America, a study conducted by Chithriki et al. (2002) examined the vertebral level of AA bifurcation, and it included the MRI for 441 patients (*n* = 205 males and 236 females) aged between 15 and 95 years [33]. The most common level of AA bifurcation was found to be L4 in 295 patients (67.00%), while the least common level was L5 in 12 patients (3%) [33].

**Figure 7:**
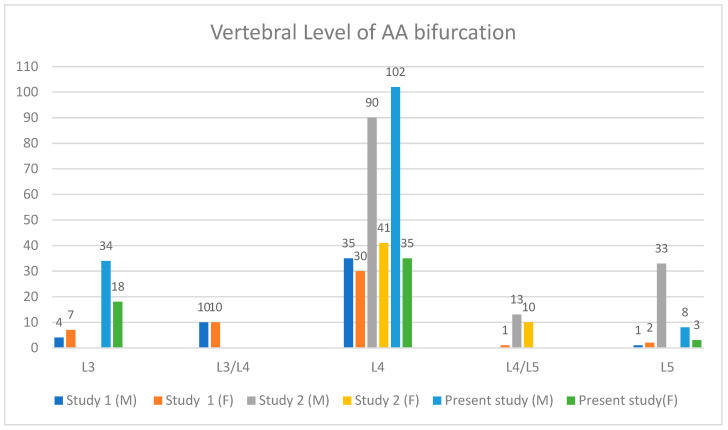
Vertebral level at which the AA bifurcated as reported by two earlier studies [32,34] and the present study (showing number of cases).

A cadaveric study was conducted in Thailand by Khamanarong et al. (2009) that included the cadavers of 187 subjects (*n* = 136 males, *n* = 51 females) and reported that the AA bifurcation was commonly found at the level of L4 in 131 subjects (70.10%) [34]. The least common level is the fourth lumbar intervertebral disc in 23 subjects (12.30%) [34]. Most of the studies agree that the most common level of AA bifurcation was L4. The findings of this study are comparable with the other studies, as the most common vertebral level for AA bifurcation was also found to be at the level of L4, which was the case in 137 (68.50%), while the least common vertebral level for AA bifurcation was found to be L5, which was the case in 11 patients (5.50%). Figure 7 shows the vertebral level of the AA bifurcation as reported by earlier and present studies. Supplementary Table S1 shows the comparison of the results of the present study with those of earlier studies.

In terms of structure, the ureters resemble the vessels. Furthermore, the ureter is closely attached to the posterior peritoneum, making its identification challenging, especially given the fact that the ureters can be found at almost any level in the retroperitoneum and the proximal pelvis [11]. Laparoscopic procedures involving the pelvis (i.e., pelvic lymphadenopathy) or gynecological procedures like salpingectomy put the ureters at risk. The ureters may be injured during aortic repair surgeries. There are numerous reports of ureter-related complications following aortic aneurysm repair surgeries [35,36]. Moreover, it is estimated that 8% of all iatrogenic ureteral injuries are due to vascular surgeries [37]. Even though ureteral injury during such procedures is rare, it may result in urinoma formation, infection, abscess formation, and loss of renal function [38]. Ultimately, this might result in legal consequences and unnecessary lawsuits [39]. Hence, the knowledge of the anatomy of the ureter and its relation to the abdominal and pelvic vessels is vital to prevent inadvertent injury to the ureter.

From a surgical point of view, it is important to know the exact position of the AA bifurcation to avoid vascular injuries. Aortic injuries are considered the main complication of the anterior approach to the lumbosacral spine, especially after the evolution of laparoscopy, which became the technique of choice in performing anterior lumbosacral interbody fusion. It is used in many lumbar disorders, such as lumbar lordosis and the decompression of foraminal stenosis. Since most of the anterior lumbosacral interbody fusion is done at the level of the L4-L5 or L5-S1 vertebrae [40], it is important to recruit vascular surgeons to mobilize the iliac arteries during the procedure [41]. Even though the vascular injuries are rare, accidental injury to a vessel might lead to the rapid development of hypovolemic shock, which can result in morbidity and mortality [34].

Due to the retrospective nature of the study, the authors had limited access to the data provided in the Hospital Information System. Some variables that are known to influence the vascular anatomy, such as height, weight, and body build, were not available in the Hospital Information System and, therefore, not considered in the present study. We strongly believe that future studies should consider these factors to examine how they reflect on the vascular anatomy. Additionally, patient selection was strictly based on the inclusion and exclusion criteria, irrespective of gender. Hence, the male-to-female ratio was 2.57:1, and the study was conducted at a single center, which may limit the generalizability of the results. Our study was designed to characterize baseline vascular anatomy in a young, healthy cohort, which we acknowledge does not reflect the pathological or age-related changes seen in aneurysm-prone populations. While this limits direct clinical applicability in older patients, we believe establishing normative data is a necessary step before pathological comparisons can be made.

## 5. Conclusions

The present radiological study was a humble attempt to highlight the clinical importance of diameter, level of bifurcation of AA, and the anatomical relation between AA and the ureter. In conclusion, the diameter of the right and left CIA was found to be wider in males compared to females, while the angle of aortic bifurcation was wider in females compared to males. A wider diameter and angle of the CIA may alter the hemodynamics of the blood vessel. Hemodynamics of the blood vessel plays an important role in the development of atherosclerosis and aneurysm.

In the present study, the aortic bifurcation was mainly at the level of the L4 vertebra, while the least common level of bifurcation was the L5 vertebra. There may be various factors that contribute to the level of aortic bifurcation.

Understanding the anatomical relation between the AA and ureters might be important to check for any inadvertent ureteral injury during aortic and pelvic surgeries. It is important to know the distance between the bifurcation of AA to the point where the ureter crosses the iliac artery for diagnostic interventions and safe ligation of the blood vessels.

The clinical implications of the variations may be important for preoperative planning and performing surgeries such as hysterectomy, the treatment of ureteric strictures, stenting in the common iliac artery, anastomosis of the iliac vessels, the removal of lymph nodes and tumors, and the repair of aneurysms.

## Figures and Tables

**Table 1:** Mean diameter of the right and left CIA proximal to the point of bifurcation (mean ± SD in mm).

Artery	≤26 Years (*n* = 52 Patients)	27–34 Years (*n* = 61 Patients)	35–42 Years (*n* = 60 Patients)	≥43 Years (*n* = 27 Patients)	*p*-Value
Right CIA	9.07 ± 1.58	9.14 ± 1.12	9.05 ± 0.99	9.06 ± 1.19	>0.05
Left CIA	9.12 ± 1.47	9.22 ± 0.91	9.07 ± 1.12	9.00 ± 1.27	>0.05

**Table 2:** Mean distance between the AA bifurcation and the point where the ureter crossed the ipsilateral iliac arteries among four age groups (mean ± SD in mm).

Side of the Blood Vessel	≤26 Years	27–34 Years	35–42 Years	≥43 Years	*p*-Value
Right	60.71 ± 12.31	56.75 ± 12.86	61.80 ± 11.40	58.96 ± 15.65	>0.05
Left	59.25 ± 11.85	58.07 ± 10.14	62.33 ± 10.14	60.96 ± 14.91	>0.05

**Table 3:** Vertebral level of AA bifurcation in males and females.

Vertebral Level	Males	Females
L3	34 (23.61%)	18 (32.14%)
L4	102 (70.83%)	35 (62.50%)
L5	8 (5.56%)	3 (5.36%)
Total	144 (100%)	56 (100%)

## Data Availability

The data presented in this study are available on request from the corresponding author.

## Supplementary Materials

The following supporting information can be downloaded at: https://www.mdpi.com/article/10.3390/diagnostics15172167/s1, Table S1: Vertebral level at which the abdominal aorta bifurcated as reported by three studies *n* (%).

## Abbreviations

AA	Abdominal aorta
CIA	Common iliac arteries
CT	Computed Tomography
EIA	External iliac arteries
K-S	Kolmogorov–Smirnov
MRI	Magnetic Resonance Imaging
SQUH	Sultan Qaboos University Hospital
