# Supplementary material for: Level of Abdominal Aorta Bifurcation and Its Relation to the Ureter: A Radiological Study with Clinical Implications
Source: Diagnostics (Basel). 2025 Aug 26;15(17):2167. doi: 10.3390/diagnostics15172167 (PMC12427873; doi:10.3390/diagnostics15172167)
Supplement: Supplementary file 1 [file diagnostics-15-02167-s001.zip › diagnostics-3833970-supplementary.pdf]

Supplementary Table 1. Vertebral level at which the abdominal aorta bifurcated as reported by three studies n (%).

	**Cited studies**					
**Vertebral level**	Khamanarong *et al* (2009) [36]		Khader *et al* (2022) [34]		Present study	
	Male	Female	Male	Female	Male	Female
**L3**	-	-	4 (8.0)	7 (14.0)	34 (23.6)	18 (32.1)
**L3/L4**	-	-	10 (20.0)	10 (20.0)	-	-
**L4**	90 (66.2)	41 (80.4)	35 (70.0)	30 (60.0)	102 (70.8)	35 (62.5)
**L4/L5**	13 (9.6)	10 (19.6)	-	1 (2.0)	-	-
**L5**	33 (24.3)	0 (0)	1 (2.0)	2 (4.0)	8 (5.6)	3 (5.4)
**Total**	136 (100)	51(100)	50 (100)	50 (100)	144 (100)	56 (100)
